# Automatic Cardiac Structure Contouring for Small Datasets with Cascaded Deep Learning Models

**DOI:** 10.1007/s10916-022-01810-6

**Published:** 2022-03-25

**Authors:** L. B. van den Oever, D. S. Spoor, A. P. G. Crijns, R. Vliegenthart, M. Oudkerk, R. N. J. Veldhuis, G. H. de Bock, P. M. A. van Ooijen

**Affiliations:** 1 Department of Radiation Oncology, University of Groningen, University Medical Center Groningen, Hanzeplein 1, 9713GZ Groningen, The Netherlands; 2 Faculty of Medical Sciences, University of Groningen, Groningen, The Netherlands; 3 Department of Radiology, University of Groningen, University Medical Center Groningen, Groningen, Netherlands; 4 Department of Electrical Engineering, Computer Science and Mathematics, University of Twente, Drienerlolaan 5, 7522 NB Enschede, The Netherlands; 5 Department of Epidemiology, University of Groningen, University Medical Center Groningen, Hanzeplein 1, 9713GZ Groningen, The Netherlands; 6 Present Address: Department of Radiology and Nuclear Medicine, Radboud University Medical Center, Nijmegen, The Netherlands

**Keywords:** Artificial Intelligence, Heart, X-ray computed tomography, Structure segmentation

## Abstract

Cardiac structure contouring is a time consuming and tedious manual activity used for radiotherapeutic dose toxicity planning. We developed an automatic cardiac structure segmentation pipeline for use in low-dose non-contrast planning CT based on deep learning algorithms for small datasets. Fifty CT scans were retrospectively selected and the whole heart, ventricles and atria were contoured. A two stage deep learning pipeline was trained on 41 non contrast planning CTs, tuned with 3 CT scans and validated on 6 CT scans. In the first stage, An InceptionResNetV2 network was used to identify the slices that contained cardiac structures. The second stage consisted of three deep learning models trained on the images containing cardiac structures to segment the structures. The three deep learning models predicted the segmentations/contours on axial, coronal and sagittal images and are combined to create the final prediction. The final accuracy of the pipeline was quantified on 6 volumes by calculating the Dice similarity coefficient (DC), 95% Hausdorff distance (95% HD) and volume ratios between predicted and ground truth volumes. Median DC and 95% HD of 0.96, 0.88, 0.92, 0.80 and 0.82, and 1.86, 2.98, 2.02, 6.16 and 6.46 were achieved for the whole heart, right and left ventricle, and right and left atria respectively. The median differences in volume were -4, -1, + 5, -16 and -20% for the whole heart, right and left ventricle, and right and left atria respectively. The automatic contouring pipeline achieves good results for whole heart and ventricles. Robust automatic contouring with deep learning methods seems viable for local centers with small datasets.

**Supplementary Information:**

The online version contains supplementary material available at 10.1007/s10916-022-01810-6.

## Introduction

**Fig. 1:**
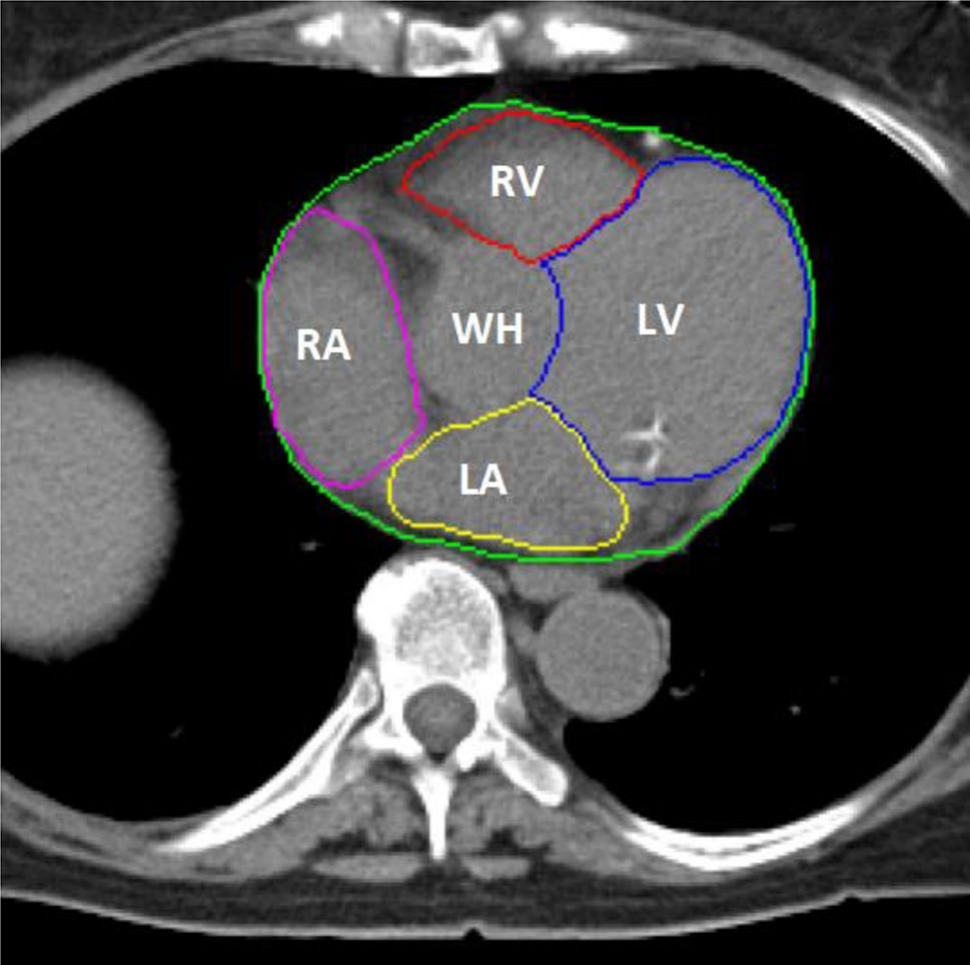
CT image with contours of the investigated structures as made by the experienced cardiac radiologist and physician assistant specialized in breast cancer: whole heart(WH) (green), left ventricle (LV) (blue), right ventricle (RV) (red), left atrium (LA) (yellow), right atrium (RA) (purple)

Cardiac image contouring is an important first step in many diagnostic and radiotherapy planning processes. Quantitative metrics can be extracted by delineating the structures of the heart, i.e., the myocardium, left and right ventricle (LV & RV) and the left and right atrium (LA & RA) (Fig. 1). These contours and measurements can give insight in the status of the heart [1], can be used to plan radiation therapy with minimal radiation toxicity [2], and can be an intermediate step in other image processing pipelines, such as automatic coronary artery calcium scoring [3]. Contouring is manually time consuming work and, therefore, finding methods to automate this process are often investigated [4, 5].

Most automated segmentation methods are based on the use of atlases. Atlas-based segmentation requires long registration times to be accurate [4]. Current advances in deep learning methods may allow for quicker and more accurate contouring and for contouring of cardiac substructures on low-dose non-contrast CT scans, such as the planning CT, instead of a contrast-enhanced CT.

However, deep learning also comes with its own disadvantages, such as the requirement for large datasets and extensive external validation. The European Commission has stated in their proposal for laying down harmonized rules on AI that AI systems should be evaluated on datasets that have appropriate statistical properties regarding the cohorts they are intended to be used on [6]. However, international high quality datasets are difficult to acquire due to privacy laws described in the same proposal. Training and evaluating deep learning methods in local centers might currently be the way forward for implementation of AI algorithms. Local centers could train robust architectures on their own data, circumventing privacy issues, and ensuring automatic validation is done on their own cohort.

The aim of this project was to develop an automated cardiac contouring pipeline for use in low-dose non-contrast CT scans in local centers with a small dataset with deep learning methods.

## Materials and Methods

Our work is part of the development process of an automated coronary artery calcium scoring algorithm [3]. Creating contours of the whole heart and the ventricles and atria may reduce the number of false positive calcium spots situated in the lungs and help identify valvular calcium. A two-stage approach with 2D neural networks was used to deal with the data imbalance problem of the heart only occupying little space in a CT volume. The first stage identified slices containing the heart so that the second stage only needs to be trained on slices with heart. The second stage segmented the whole heart, the LV and RV, and the LA and RA.

### Dataset

Fifty planning CT scans of patients with breast cancer were selected for this study. These scans contained no clinically relevant abnormalities; the scans were part of radiotherapeutic treatment at the University Medical Center Groningen [7].

The CT examinations were performed with a Somatom Sensation Open CT system (Siemens Healthineers, Erlangen, Germany), without iodine contrast agent. Field of view was set at 500 mm and 512 × 512 pixels with a thickness of 3 (N = 29) or 5 (N = 21) mm. No interpolation was used to reconstruct the volumes. The CT images were converted to NUMPY format, the contrast was adjusted to the mediastinum window setting (W:350 HU, L:50 HU). No further pre-processing was done on the images.

An experienced cardiac radiologist and physician assistant specialized in breast cancer created manual contours of the whole heart, LV and RV, and LA and RA, based on the atlas of Feng et al. [8] by general consensus. This work was performed in Mirada RTx (v1.6 Mirada Medical Ltd., Oxford, UK).

**Fig. 2:**
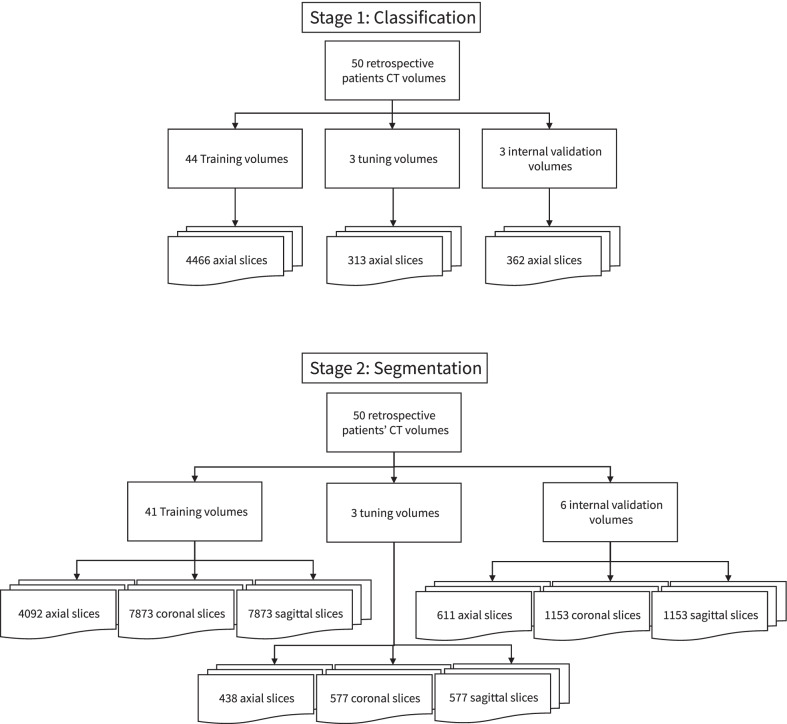
Summary of the datasplit of the first and second stage training, tuning and validation

The dataset was split in 44 training volumes, 3 tuning volumes and 3 internal validation volumes. All 2D slices were extracted from these volumes for the first stage. For the second stage, only the images that contained relevant heart contours were used. We also added 3 slices of liver without heart contours to improve the networks’ recognition of liver areas. The dataset was resplit into 41 training volumes, 3 tuning volumes and 6 internal validation volumes for the second stage by moving 3 3-mm slice thickness patient volumes from the training dataset to the internal validation dataset. The splitting of the dataset was done to get a 50–50 division in the validation volumes between 3 and 5 mm slice thickness scans. An overview of the datasplit is shown in Fig. 2.

### Network Architectures

The contouring pipeline consisted of two stages. The first stage was a classifier that classifies axial slices into slices that contain the heart and slices without heart. The architecture was InceptionResNetV2 and is, as the name implies, a hybrid architecture combining the strengths of the Inception and Resnet architectures. This network is 164 layers deep and still trainable thanks to the addition of residual layers in the Inception blocks[9]. The network was pretrained on the ImageNet Large Scale Visual Recognition Challenge containing around 1.2 million images with 1000 categories of day-to-day objects[9]. This challenge evaluated models for object detection and image classification. The model was, therefore, already familiar with standard shapes and this makes training of the model easier. The batches consisted of 15 CT slices, the maximum amount of epochs was set to 50 with early stopping based on the F1 metric of the tuning set to prevent false negatives. Early stopping based on the F1 metric was achieved after 89 epochs. The loss function was binary cross entropy and the learning rate was set to 10e^−4^ with Adamax as optimizer.

The second stage consisted of three Unet3 + neural networks[10]. These neural networks worked parallel to each other. One segmented the five structures on axial slices whereas the other two only segment the whole heart on coronal and sagittal slices to improve the segmentations of the lower and upper boundaries of the heart. The output of these three neural networks was combined into a volume by multiplication of the binary masks for the final result. The batch size was set to 5 slices, with Adamax with a learning rate of 10e^−3^, beta_1 of 0.9, beta_2 of 0.999 and an epsilon of 1e^−7^ as an optimizer. The loss function used was a hybrid loss of the sum of the dice coefficient loss function and the focal loss function (**Eq. ****1**). The focal loss function was weighted twice to help improve accuracy on slices that were difficult to contour.

1 $${\mathcal{L}}_{hybrid}=2*{\mathcal{L}}_{focal}+{\mathcal{L}}_{dice}$$

The architectures of both stages were implemented in Python 3.6 with Keras[11] with TensorFlow backend.

### Metrics

Cohen’s kappa was used as a metric for the accuracy of the first stage classifier. The Dice similarity coefficient[12] (DC) and 95% Hausdorff distance[13] (95% HD) are used as metrics to quantify the overlap with and distance of the contours when compared to the ground truth contours[14]. The ratio between the manual and predicted volumes of each structure is used as a metric for clinical implications of using the proposed method.

## Results

The first classifier for selection of slices containing the heart was very accurate with an accuracy of 99% and an agreement with Cohen’s kappa of 0.98 between the ground truth and the classifier. Table 1 shows the confusion matrix.

**Table 1:** Confusion matrix of the internal validation dataset of the classifier for axial slices containing the heart

		**Prediction (Slices)**	
		**Without heart**	**With heart**
**Ground Truth (Slices)**	Without heart	268	1
	With heart	2	90

**Fig. 3:**
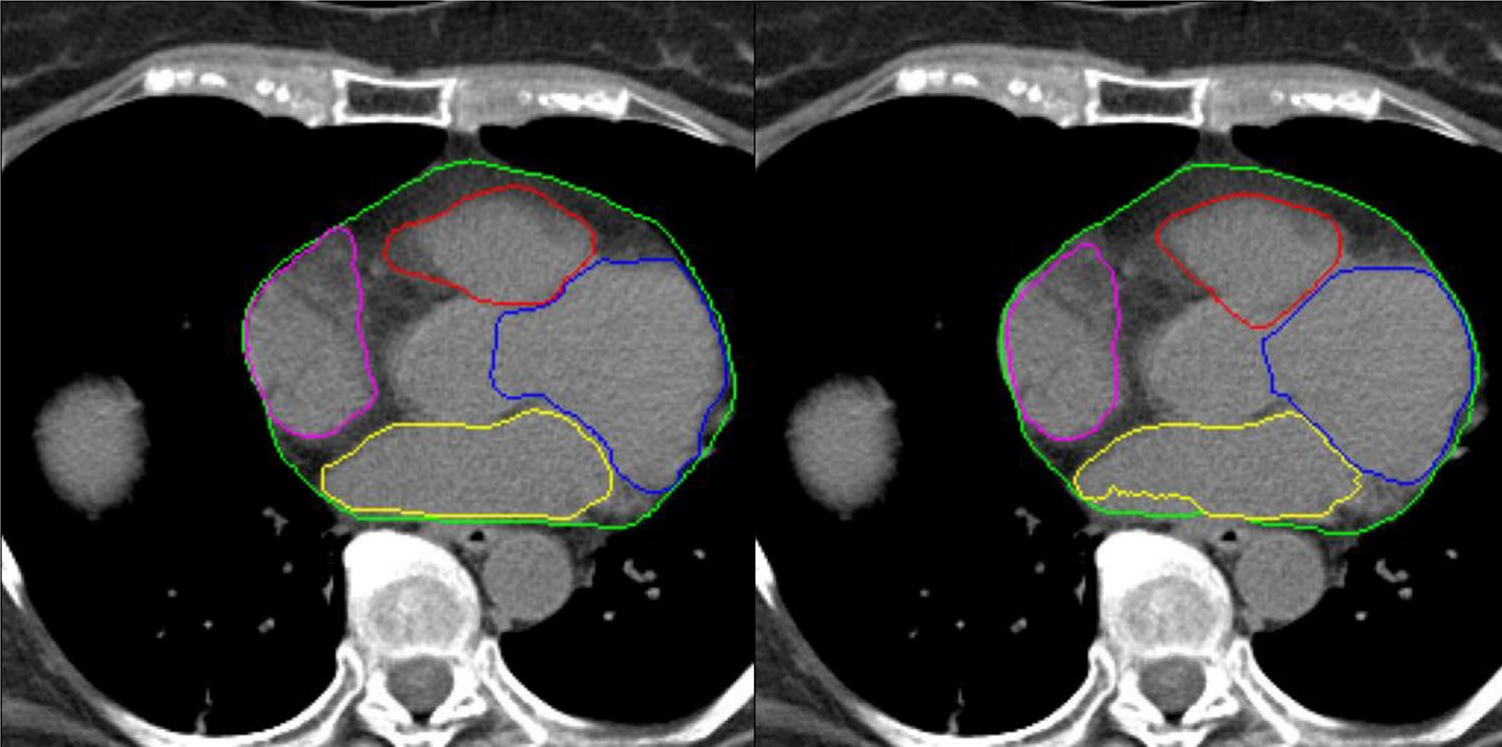
Example of annotated contours (left) and predicted contours (right). Whole heart(WH) (green), left ventricle (LV) (blue), right ventricle (RV) (red), left atrium (LA) (yellow), right atrium (RA) (purple)

The second stage 2.5D neural networks achieved a median DC of 0.96, 0.88, 0.92, 0.82 and 0.80 for the whole heart, RV, LV, LA and RA contours. 95% HDs were 1.86, 2.98, 2.02, 6.46 and 6.16 mm for the whole heart, RV, LV, LA and RA contours. Volume ratios between the ground truth volumes and the predicted volumes were 0.96, 0.99, 1.05, 0.80 and 0.84 for the whole heart, RV, LV, LA and RA respectively (Table 2). An average example of the contours can be seen in Fig. 3. More detailed results of each validation dataset can be found in supplementary table 1. The entire contouring pipeline takes under 30 s to process an entire volume even though the process is currently unoptimized. We speculate that the entire process could take well under 10 seconds if optimized.

**Table 2:** Median results of the 2.5D neural networks for cardiac structure segmentation. The first and third quartiles are given in parentheses

	**Volume ratio**	**Dice Coefficient**	**95% Hausdorff Distance (mm)**
WH	0.96 (0.95 – 0.97)	0.96 (0.94 – 0.96)	1.86 (1.40 – 3.01)
RV	0.99 (0.98 – 1.02)	0.88 (0.87 – 0.90)	2.98 (2.46 – 3.33)
LV	1.05 (1.00 – 1.07)	0.92 (0.90 – 0.93)	2.02 (1.98 – 3.72)
LA	0.80 (0.73 – 0.84)	0.82 (0.80 – 0.84)	6.46 (3.81 – 8.93)
RA	0.84 (0.79 – 0.91)	0.80 (0.77 – 0.83)	6.16 (3.42 – 7.17)

*WH* whole heart, *RV* right ventricle, *LV* left ventricle, *LA* left atrium, *RA* right atrium

## Discussion

We show that our proposed deep learning method enables automatic contouring of heart chambers in low-dose non-contrast radiotherapy planning CTs with only a small dataset, so can be used in local centers. The use of a local small dataset would ensure that the algorithms work on the local population, circumventing both privacy issues for acquiring large datasets and biases associated with such large datasets.

Manual contouring of CT scans is tedious and time consuming, and our method could provide the first contours that medical technicians or radiologists then only need to adjust. This method can be used for radiation therapy planning, calculating cardiac volumes, or improving existing cardiac image processing pipelines used for automatically detecting coronary calcifications. The pipeline creates contours in under 30 s. This means that the software could easily be implemented right after the scans have been made and before the contours are adjusted by technicians.

High accuracy can be reached by reducing the class imbalance between the slices with cardiac structures and without. The contours of the whole heart and ventricles show small changes in volumes between the ground truth and predictions with median volume ratios of 0.96, 0.99 and 1.05 and median DCs of 0.96, 0.88 and 0.92 and 95% HDs around 3 mm.

Similar work on cardiac contouring has been done both on non-contrast and contrast CT with both atlas and deep learning based methods (Table 3). Choi et al. developed a deep learning method to create contours of cardiac structures according to the ESTRO guidelines [15]. They compare their method to two atlas based commercial software methods. We achieved slightly better results on the whole heart and ventricles with higher DCs and, more importantly, lower 95% HDs. The correlation between 95% HD and time needed to adjust contours might be better than the correlation between the DC and time needed to adjust. The DC is sensitive to the size of a segmentation, which is why distance based metrics might be clinically more relevant. 95% HD is more sensitive to the accuracy of the contours itself. Choi et al. achieve lower DCs on the atria compared to the ventricles, which we also see in our work. It is also shown that their results with the deep learning based method achieve more robust and more consistent results than the commercial atlas based methods. Hopefully, this might also translate to our results, since our method has similar accuracies.

**Table 3:** Comparison between the proposed method and recent other methods. Values are the mean values of the metric with the standard deviation. Stated after the authors name is the number of datasets in the training/test set, whether non contrast CT (NCCT) or contrast CT (CCT) were used and whether the method was based on deep learning (DL) algorithms or atlas based algorithms. In the case of atlas based methods, N indicates the number of volumes used to create the atlas/the number tested on. **Bold** values indicate the highest score of all the NCCT projects

	**Choi et al. **[15] **(N = 35/14) (NCCT)** **(DL based)**		**Harms et al. **[16]** (N = 30/15) (CCT & NCCT) (DL based)**		**Jung et al. **[17]** (N = 29/1) (NCCT)** **(Atlas based)**		**Luo et al. **[18] **(N = 12/49) (NCCT)** **(Atlas based)**		**Proposed method (N = 41/6) (NCCT) (DL based)**	
	***DC***	***95% HD (mm)***	***DC***	***95% HD (mm)***	***DC***	***95% HD (mm)***	***DC***	***95% HD (mm)***	***DC***	***95% HD (mm)***
**WH**	0.95 ± 0.01	2.39 ± 0.47	0.96 ± 0.03	6.00 ± 5.73	**0.97 ± 0.01**	NR	0.95 ± 0.04	NR	0.95 ± 0.01	**2.22 ± 0.97**
**RV**	0.86 ± 0.04	6.15 ± 2.39	0.92 ± 0.03	4.43 ± 1.95	0.69 ± 0.08	NR	0.87 ± 0.10	NR	**0.88 ± 0.02**	**2.88 ± 0.67**
**LV**	0.87 ± 0.03	4.52 ± 1.65	0.96 ± 0.01	3.80 ± 1.46	0.80 ± 0.06	NR	0.91 ± 0.06	NR	**0.92 ± 0.01**	**3.27 ± 2.04**
**LA**	0.78 ± 0.04	**4.77 ± 2.18**	0.90 ± 0.08	5.17 ± 4.39	0.67 ± 0.10	NR	**0.89 ± 0.05**	NR	0.82 ± 0.03	7.35 ± 4.61
**RA**	0.85 ± 0.04	**4.03 ± 1.20**	0.94 ± 0.02	3.44 ± 1.12	0.68 ± 0.10	NR	**0.86 ± 0.12**	NR	0.81 ± 0.05	6.06 ± 3.32

*NCCT* non contrast CT, *CCT* contrast CT, *DL* deep learning, *WH* whole heart, *RV* right ventricle, *LV* left ventricle, *LA* left atrium, *RA* right atrium, *NR* not reported, *HD* Hausdorff distance, *DC* dice similarity coefficient

Harms et al. created a processing pipeline with 5 cascaded neural networks trained and validated on both contrast and non-contrast scans [16]. Their results show slightly better accuracy for the whole heart and ventricles, and significantly better accuracy for the atria. This is probably due to the increased complexity and depth of their method and the use of contrast scans, which are easier to annotate, therefore creating more accurate contours, and easier to contour for deep learning based methods due to increased contrast and ECG-triggering, and, therefore, clearer boundaries between structures.

Jung et al. and Luo et al. used atlas based contouring methods for creating cardiac contours on non-contrast CT scans [17, 18]. Luo et al. used non contrast 4D CT scans, averaged between respiratory cycles, to achieve similar results to our work. Interestingly, they show that very little modifications were necessary for the predicted contours and the differences in dosimetry were not significant. It will be interesting to investigate if the proposed method also has similar dose predictions compared to the ground truth. Therefore, future work will be done in the form of qualitative evaluations of our work.

### Limitations

**Fig. 4:**
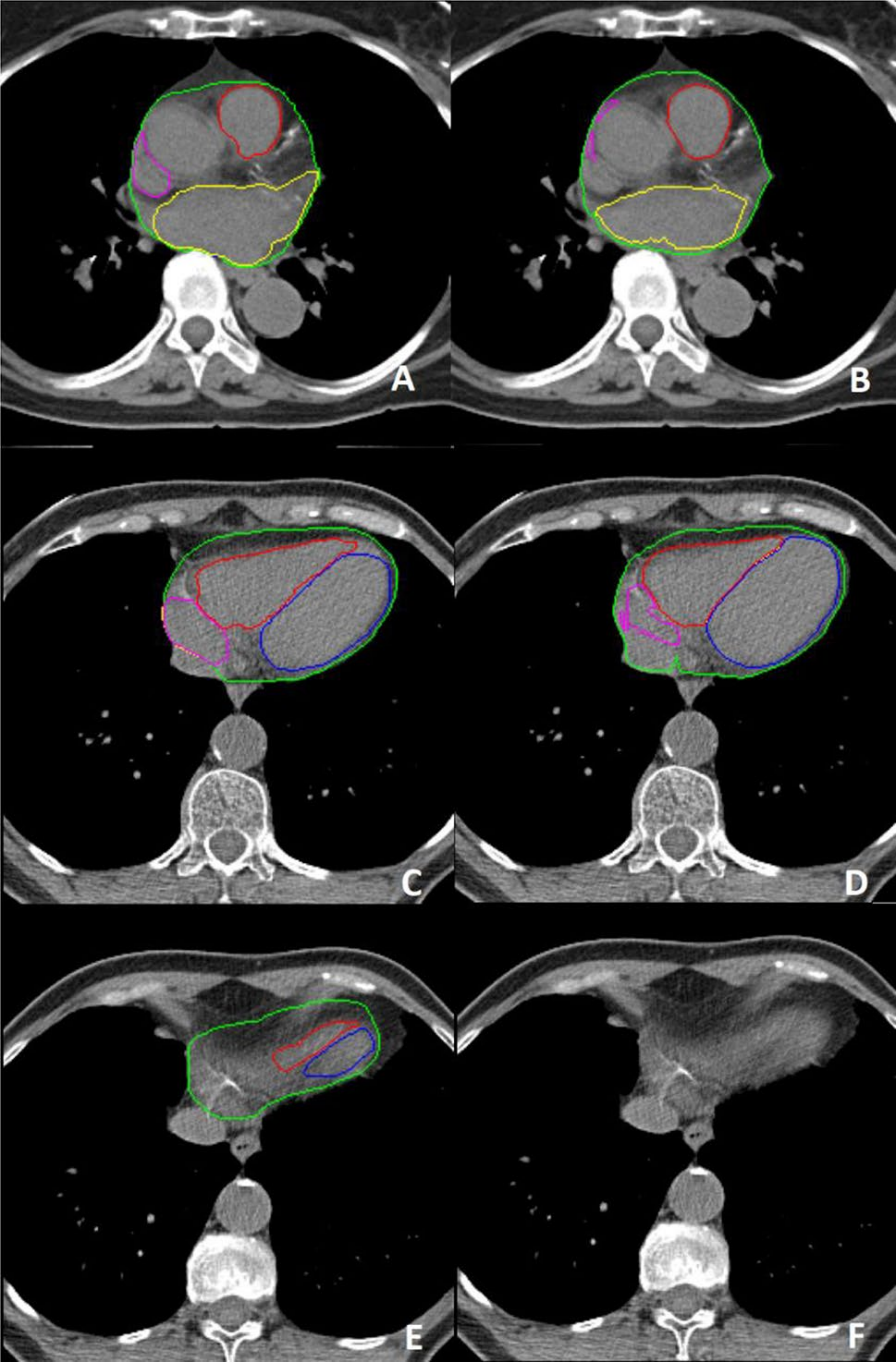
Example of poor segmentations in the cranial (**A** & **B**) and caudal (**C** - **F**) part of the heart with on the left the ground truth and on the right the predicted contours by the deep learning pipeline. Whole heart(WH) (green), left ventricle (LV) (blue), right ventricle (RV) (red), left atrium (LA) (yellow), right atrium (RA) (purple). Note the largely missed right atrium (**B** & **D**) and the overestimation of the left atrium (**B**). Image F shows the most caudal slice being misclassified as containing no cardiac structures

This work has a number of limitations. The deep learning pipeline struggles with slices that it has trained on very little, i.e. the caudal and cranial parts of the heart (Fig. 4). Most of the errors occur in the caudal and cranial 5% of the slices. The networks also have difficulty near the apex of the heart, where the contrast between the liver and the heart is minimal. Oversegmentation is prevented by the use of the first stage classifier, however, localization of the heart can be difficult in the few slices that still have liver and where the shape of the heart is barely recognizable.

The contouring pipeline underperforms for the atria when compared to the ventricles and whole heart contours. This is also seen in the recent works from other authors as seen in Table 3. This results in significant volume differences between ground truth and predicted volumes. This might be due to the atria having a significantly smaller volume than the ventricles and, therefore, have a higher class imbalance within the image problem. This problem must first be solved before implementation into clinical practice.

The contours were created on non-contrast scans with relatively thick slices and cardiac motion due to no ECG triggering, which can make it difficult to find borders between structures with the human eye. This might lead to mismatches in contouring. If this is the case, it might be harder to achieve higher accuracy, since the pipeline is quite consistent in its contouring, whereas humans might be less consistent. Therefore, the ground truth might have differences in contouring methods when compared to the predicted contours, leading to lower validation accuracy. The ideal solution for this would be to have contoured and registered contrast and non-contrast scans and train the deep learning methods on the contrast scans. Harms et al. showed even higher accuracy on neural networks trained on both contrast and non-contrast scans in different patients, so that might also be a possibility.

### Implications

This work shows that acceptable accuracy can be achieved for automated contouring software based on deep learning with only a limited amount of datasets. This implies that hospitals could use their own data to create deep learning tools for their own patient cohort with their specific CT scan protocol. This would circumvent the need for very generalized neural networks and ensures that validation of such software is done on a hospital’s own patient/scan protocol composition. In this case, most radiotherapy departments would have contouring data available, so could easily train this method on their own data if hardware to do so is available.

The whole heart contours are shown to be very accurate. Therefore, the method described in this paper could be used as an intermediate step, such as automatic coronary artery calcium scoring. Any false positive calcium spots in the lungs or other areas surrounding the heart could be removed with the use of the whole heart segmentation. These spots are usually more than a centimeter removed from the heart. Therefore, the 95% HD of under 2 mm should be adequate.

## Supplementary Information

Below is the link to the electronic supplementary material.

Supplementary file1 (DOCX 15 KB)

## References

[1] Roos CTG, van den Bogaard VAB, Greuter MJW, Vliegenthart R, Schuit E, Langendijk JA, van der Schaaf A, Crijns APG, Maduro JH (2018). Is the coronary artery calcium score associated with acute coronary events in breast cancer patients treated with radiotherapy?. Radiotherapy and Oncology..

[2] Haq R, Hotca A, Apte A, Rimner A, Deasy JO, Thor M (2020). Cardio-pulmonary substructure segmentation of radiotherapy computed tomography images using convolutional neural networks for clinical outcomes analysis, Physics and Imaging in Radiation. Oncology..

[3] van den Oever LB, Cornelissen L, Vonder M, Xia C, van Bolhuis JN, Vliegenthart R, Veldhuis RNJ, de Bock GH, Oudkerk M, van Ooijen PMA (2020). Deep learning for automated exclusion of cardiac CT examinations negative for coronary artery calcium. European Journal of Radiology..

[4] Cardenas CE, Yang J, Anderson BM, Court LE, Brock KB (2019). Advances in Auto-Segmentation. Seminars in Radiation Oncology..

[5] Sharp G, Fritscher KD, Pekar V, Peroni M, Shusharina N, Veeraraghavan H, Yang J (2014). Vision 20/20: Perspectives on automated image segmentation for radiotherapy. Medical Physics..

[6] European Commission, Proposal for a Regulation laying down harmonised rules on artificial intelligence. (2021) 1–108. https://digital-strategy.ec.europa.eu/en/library/proposal-regulation-laying-down-harmonised-rules-artificial-intelligence.

[7] H.P. Van der Laan, W. V. Dolsma, J.H. Maduro, E.W. Korevaar, J.A. Langendijk, Dosimetric consequences of the shift towards computed tomography guided target definition and planning for breast conserving radiotherapy. Radiation Oncology. 3 (2008). 10.1186/1748-717X-3-6. 10.1186/1748-717X-3-6 PMC2270860 18237427

[8] Feng M, Moran JM, Koelling T, Chughtai A, Chan JL, Freedman L, Hayman JA, Jagsi R, Jolly S, Larouere J, Soriano J, Marsh R, Pierce LJ (2011). Development and validation of a heart atlas to study cardiac exposure to radiation following treatment for breast cancer. International Journal of Radiation Oncology Biology Physics..

[9] C. Szegedy, S. Ioffe, V. Vanhoucke, A.A. Alemi, Inception-v4, inception-ResNet and the impact of residual connections on learning. In 31st AAAI Conference on Artificial Intelligence, AAAI 2017, AAAI press, 2017: pp. 4278–4284.

[10] H. Huang, L. Lin, R. Tong, H. Hu, Q. Zhang, Y. Iwamoto, X. Han, Y.-W. Chen, J. Wu, UNet 3+: A Full-scale connected UNet for medical image segmentation, ICASSP, IEEE International Conference on Acoustics, Speech and Signal Processing - Proceedings. 2020-May (2020) 1055–1059.

[11] F. Chollet, Keras, (2015). https://github.com/fchollet/keras.

[12] Dice LR (1945). Measures of the Amount of Ecologic Association Between Species. Ecology..

[13] Huttenlocher DP, Klanderman GA, Rucklidge WJ (1993). Comparing Images Using the Hausdorff Distance. IEEE Transactions on Pattern Analysis and Machine Intelligence..

[14] Maier-Hein L, Eisenmann M, Reinke A, Onogur S, Stankovic M, Scholz P, Arbel T, Bogunovic H, Bradley AP, Carass A, Feldmann C, Frangi AF, Full PM, van Ginneken B, Hanbury A, Honauer K, Kozubek M, Landman BA, März K, Maier O, Maier-Hein K, Menze BH, Müller H, Neher PF, Niessen W, Rajpoot N, Sharp GC, Sirinukunwattana K, Speidel S, Stock C, Stoyanov D, Taha AA, van der Sommen F, Wang C-W, Weber M-A, Zheng G, Jannin P, Kopp-Schneider A (2018). Why rankings of biomedical image analysis competitions should be interpreted with care. Nature Communications..

[15] Choi MS, Choi BS, Chung SY, Kim N, Chun J, Kim YB, Chang JS, Kim JS (2020). Clinical evaluation of atlas- and deep learning-based automatic segmentation of multiple organs and clinical target volumes for breast cancer. Radiotherapy and Oncology..

[16] Harms J, Lei Y, Tian S, McCall NS, Higgins KA, Bradley JD, Curran WJ, Liu T, Yang X (2021). Automatic delineation of cardiac substructures using a region-based fully convolutional network. Medical Physics..

[17] Jung JW, Lee CCC, Mosher EG, Mille MM, Yeom YS, Jones EC, Choi M, Lee CCC (2019). Automatic segmentation of cardiac structures for breast cancer radiotherapy, Physics and Imaging in Radiation. Oncology..

[18] Luo Y, Xu Y, Liao Z, Gomez D, Wang J, Jiang W, Zhou R, Williamson R, Court LE, Yang J (2019). Automatic segmentation of cardiac substructures from noncontrast CT images: accurate enough for dosimetric analysis?. Acta Oncologica..

