# Supplementary material for: Automatic Cardiac Structure Contouring for Small Datasets with Cascaded Deep Learning Models
Source: J Med Syst. 2022 Mar 25;46(5):22. doi: 10.1007/s10916-022-01810-6 (PMC8956542; doi:10.1007/s10916-022-01810-6)
Supplement: Supplementary file 1 — Supplementary file1 (DOCX 15 KB) [file 10916_2022_1810_MOESM1_ESM.docx]

|  | Volume Ratio | | | | | Dice Coefficient | | | | | | 95% Hausdorff Distance (mm) | | | | | |
| --- | --- | --- | --- | --- | --- | --- | --- | --- | --- | --- | --- | --- | --- | --- | --- | --- | --- |
| Patient ID | WH | RV | LV | LA | RA | WH | RV | LV | LA | RA | median | WH | RV | LV | LA | RA | median |
| 11 | 1.00 | 1.04 | 1.06 | 0.84 | 0.78 | 0.96 | 0.85 | 0.90 | 0.82 | 0.75 | 0.85 | 1.34 | 3.8 | 2.04 | 9.54 | 12.0 | 3.80 |
| 12 | 0.96 | 0.99 | 0.95 | 0.91 | 1.06 | 0.97 | 0.88 | 0.92 | 0.88 | 0.81 | 0.88 | 2.15 | 3.13 | 2.00 | 3.13 | 7.48 | 3.13 |
| 13 | 0.94 | 1.03 | 1.07 | 0.75 | 0.83 | 0.96 | 0.88 | 0.93 | 0.81 | 0.78 | 0.88 | 1.56 | 2.83 | 1.97 | 16.2 | 6.07 | 2.83 |
| 14 | 0.97 | 0.92 | 1.13 | 0.72 | 0.67 | 0.94 | 0.86 | 0.90 | 0.77 | 0.77 | 0.86 | 1.15 | 3.39 | 4.28 | 7.08 | 6.25 | 4.28 |
| 10 | 0.96 | 0.98 | 0.98 | 0.84 | 0.93 | 0.94 | 0.90 | 0.93 | 0.84 | 0.84 | 0.93 | 3.69 | 1.79 | 1.93 | 2.33 | 2.00 | 2.00 |
| 1 | 0.94 | 0.98 | 1.04 | 0.72 | 0.85 | 0.95 | 0.90 | 0.91 | 0.80 | 0.89 | 0.90 | 3.30 | 2.33 | 7.42 | 5.84 | 2.53 | 3.30 |
|  |  |  |  |  |  |  |  |  |  |  |  |  |  |  |  |  |  |
| median | 0.96 | 0.99 | 1.05 | 0.80 | 0.84 | 0.96 | 0.88 | 0.92 | 0.82 | 0.80 | 0.88 | 1.86 | 2.98 | 2.02 | 6.46 | 6.16 | 3.22 |
| Q1 | 0.95 | 0.98 | 1.00 | 0.73 | 0.79 | 0.94 | 0.87 | 0.90 | 0.80 | 0.77 | 0.87 | 1.40 | 2.46 | 1.98 | 3.81 | 3.42 | 2.91 |
| Q3 | 0.97 | 1.02 | 1.07 | 0.84 | 0.91 | 0.96 | 0.90 | 0.93 | 0.87 | 0.83 | 0.90 | 3.01 | 3.33 | 3.72 | 8.93 | 7.17 | 3.68 |

**Supplementary Table 1**
